# 3-({4-[(2-Methyl­benzyl­idene)amino]-5-sulfanyl­idene-1*H*-1,2,4-triazol-3-yl}meth­yl)-1,3-benzoxazol-2(3*H*)-one

**DOI:** 10.1107/S1600536812051458

**Published:** 2013-01-04

**Authors:** Abdullah Aydın, Nuray Hekimoğlu, Mehmet Akkurt, Tijen Önkol, Şölen Urlu Çiçekli, Orhan Büyükgüngör

**Affiliations:** a Department of Science Education, Faculty of Education, Kastamonu University, 37200 Kastamonu, Turkey; b Department of Physics, Institute of Science and Technology, Kastamonu University, 37100 Kastamonu, Turkey; c Department of Physics, Faculty of Sciences, Erciyes University, 38039 Kayseri, Turkey; d Department of Pharmaceutical Chemistry, Faculty of Pharmacy, Gazi University, 06330 Ankara, Turkey; e Department of Physics, Faculty of Arts and Sciences, Ondokuz Mayıs University, 55139 Samsun, Turkey

## Abstract

In the title compound, C_18_H_15_N_5_O_2_S, a weak intra­molecular C—H⋯S hydrogen bond results in a small dihedral angle of 3.71 (9)° between the methyl­phenyl and triazole rings, which, in turn, form dihedral angles of 80.09 (8) and 77.32 (8)°, respectively, with the benzoxazolone mean plane. In the crystal, N—H⋯O hydrogen bonds link mol­ecules into chains along [001], and weak C—H⋯N hydrogen bonds and π–π inter­actions between the five- and six-membered rings [centroid–centroid distances = 3.5074 (11) and 3.616 (1) Å] consolidate the crystal packing.

## Related literature

For details of the synthesis, see: Urlu-Cicekli *et al.* (2012 ▶). For related structures, see: Aydın *et al.* (2005 ▶, 2012 ▶). For a MOPAC AM1 theoretical full-geometry optimization, see: Dewar *et al.* (1985 ▶); Stewart (1993 ▶).
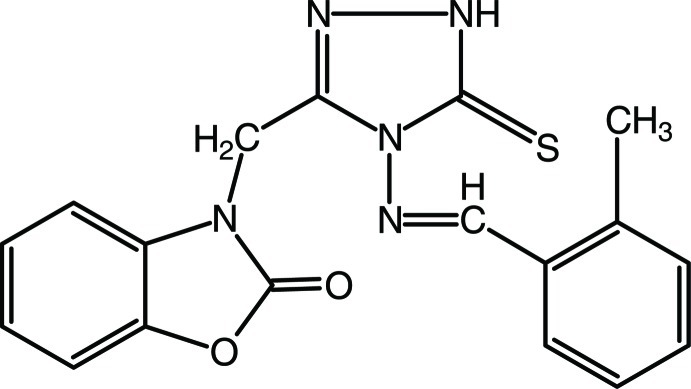


## Experimental

### 

#### Crystal data

C_18_H_15_N_5_O_2_S
*M* *_r_* = 365.42
Monoclinic, 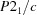

*a* = 18.0823 (13) Å
*b* = 6.4623 (4) Å
*c* = 15.1892 (11) Å
β = 100.821 (6)°
*V* = 1743.4 (2) Å^3^
*Z* = 4
Mo *K*α radiation
μ = 0.21 mm^−1^
*T* = 296 K
0.62 × 0.48 × 0.22 mm

#### Data collection

Stoe IPDS 2 diffractometer
Absorption correction: integration (*X-RED32*; Stoe & Cie, 2002 ▶) *T*_min_ = 0.881, *T*_max_ = 0.955
10084 measured reflections
3958 independent reflections
3034 reflections with *I* > 2σ(*I*)
*R*_int_ = 0.029

#### Refinement

*R*[*F* ^2^ > 2σ(*F* ^2^)] = 0.042
*wR*(*F* ^2^) = 0.110
*S* = 1.03
3958 reflections
237 parameters
H-atom parameters constrained
Δρ_max_ = 0.21 e Å^−3^
Δρ_min_ = −0.27 e Å^−3^

### 

Data collection: *X-AREA* (Stoe & Cie, 2002 ▶); cell refinement: *X-AREA*; data reduction: *X-RED32* (Stoe & Cie, 2002 ▶); program(s) used to solve structure: *SIR97* (Altomare *et al.*, 1999 ▶); program(s) used to refine structure: *SHELXL97* (Sheldrick, 2008 ▶); molecular graphics: *ORTEP-3 for Windows* (Farrugia, 1997 ▶); software used to prepare material for publication: *WinGX* (Farrugia, 1999 ▶) and *PLATON* (Spek, 2009 ▶).

## Supplementary Material

Crystal structure: contains datablock(s) global, I. DOI: 10.1107/S1600536812051458/cv5375sup1.cif

Structure factors: contains datablock(s) I. DOI: 10.1107/S1600536812051458/cv5375Isup2.hkl

Supplementary material file. DOI: 10.1107/S1600536812051458/cv5375Isup3.cml

Additional supplementary materials:  crystallographic information; 3D view; checkCIF report

## Figures and Tables

**Table 1:** Hydrogen-bond geometry (Å, °)

*D*—H⋯*A*	*D*—H	H⋯*A*	*D*⋯*A*	*D*—H⋯*A*
N3—H3*A*⋯O2^i^	0.86	2.03	2.856 (2)	162
C4—H4⋯N4^ii^	0.93	2.52	3.387 (3)	155
C11—H11⋯S1	0.93	2.48	3.2159 (18)	136

Symmetry codes: (i) 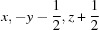; (ii) 
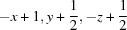
.

**Fig. 1.**
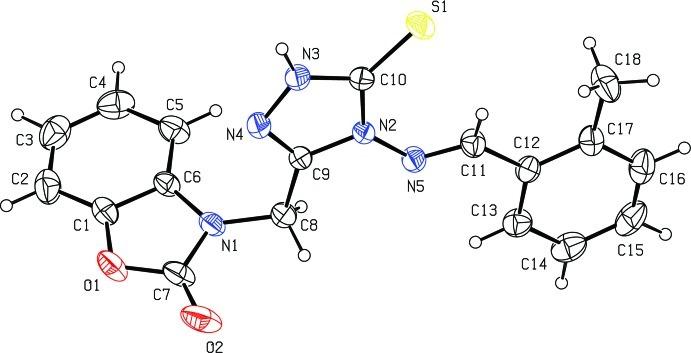
The molecule shown with the atom numbering scheme. Displacement ellipsoids for non-H atoms are drawn at the 30% probability level.

**Fig. 2.**
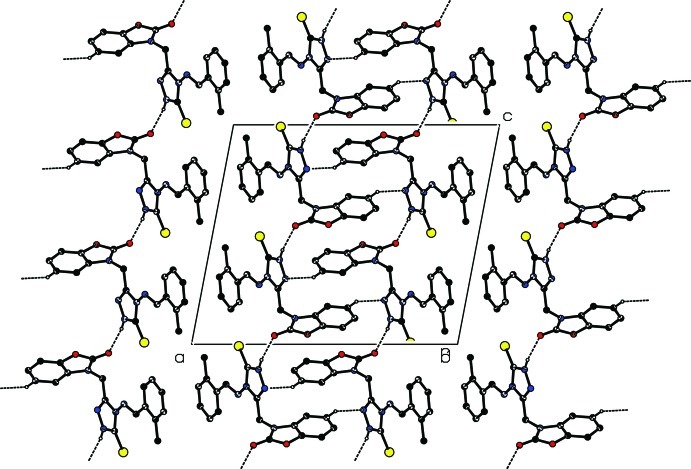
The packing and hydrogen bonding of the title compound viewed down the b axis. H atoms not involved in hydrogen bondings are omitted for the sake of clarity.

## supplementary crystallographic information

### Comment

In continuation of our studies of hybrid molecules containing 2(3*H*)- benzoxazolone fragment (Aydın *et al.*, 2005), herewith we present the title compound, (I). In (I) (Fig. 1), the 2,3-dihydro-1,3-benzoxazole ring (N1/O1/C1—C7) is essentially planar with the maximum deviation of the C7 atom from the mean plane of -0.034 (2) Å. This ring system makes dihedral angles of 77.32 (8) and 80.09 (8)°, with the 4,5-dihydro-1*H*-1,2,4-triazole ring (N2–N4/C9/C10) and the benzene ring (C12–C17), respectively. The dihedral angle between the 4,5-dihydro-1*H*-1,2,4-triazole ring and the benzene ring is 3.71 (9)°. All bond lengths and angles are comparable with those observed in similar compounds (Aydın *et al.*, 2005; 2012).

In the crystal, neighbouring molecules are linked by N—H···O and C—H···N hydrogen bonding interactions, forming a two dimensional network parallel to the (101) plane (Table 1, Fig. 2). In addition, the crystal packing is stabilized by a weak C—H···π interaction and two π-π stacking interactions [*Cg*1···*Cg*3(1 - *x*, -*y*, -*z*) = 3.5074 (11) Å and *Cg*2···*Cg*4(*x*, -1 + *y*,*z*) = 3.6160 (10) Å; where *Cg*1, *Cg*2, *Cg*3 and *Cg*4 are the centroids of the O1/C1/C6/N1/C7, N2/C9/N4/N3/C10, C1–C6 and C12–C17 rings, respectively].

Molecular orbital calculations using semi-empirical (AM1) have been carried out for the title compound with MOPAC (Dewar *et al.*, 1985; Stewart, 1993). The values of the structural parameters of the title compound obtained by the results of the theoretical calculations (based on isolated molecules) and X-ray structural determinations in the solid state are almost identical within experimental error. The calculated dipole moment of (I) is 3.243 D. The HOMO and LUMO energy levels are -8.65563 and -.31527 eV, respectively.

### Experimental

To a suspension of *o*-methylbenzaldehyde (0.0022 mol) in glacial acetic acid (3 ml), 0.002 mol [(4-amino-5-sulfanylidene-1,2,4-triazol-3-yl) methyl]-2(3*H*)-benzoxazolone was added. The reaction mixture was placed in microwave oven and irradiated for minutes changing between 15–30 min at 398 K (300 W). After completion of the reaction by monitoring with TLC, the reaction mixture was kept overnight at room temperature. The precipitate was collected by filtration, washed with water, dried, and crystallized from EtOH-acetone.

Yield, 58%, m.p.: 494–495 K. IR *v*_max_ cm^-1^, 3186, 1772, 1484, 1268. ^1^H-NMR (DMSO-d_6_) δ 14.16 (1*H*, s, NH), 10.28 (1*H*, s, =CH), 7.84 (1*H*, d, Ar—H), 7.39 (1*H*, t, Ar—H), 7.29–7.18 (4*H*, m, Ar—H, H7, H4), 7.12 (1*H*, t, H6), 7.06 (1*H*, t, H5), 5.21 (2*H*, s, CH_2_), 2.40 (3*H*, s, CH_3_). Elemental analysis: C_18_H_15_N_5_O_2_S, Calc.(%) / Found (%): C:59.16/59.38, H: 4.14/3.95, N: 19.17/19.15. (Urlu-Cicekli *et al.*, 2012).

### Refinement

H atoms were positioned geometrically, with N—H = 0.86 Å, C—H = 0.93(aromatic), 0.97(methylene) and 0.96 Å (methyl), and refined as riding with *U*_iso_(H) = 1.5*U*_eq_(C) for methyl H and 1.2*U*_eq_(C,*N*) for the others.

### Crystal data

**Table:**

C_18_H_15_N_5_O_2_S	*F*(000) = 760
*M**_r_* = 365.42	*D*_x_ = 1.392 Mg m^−^^3^
Monoclinic, *P*2_1_/*c*	Mo *K*α radiation, λ = 0.71073 Å
Hall symbol: -P 2ybc	Cell parameters from 14230 reflections
*a* = 18.0823 (13) Å	θ = 1.6–28.1°
*b* = 6.4623 (4) Å	µ = 0.21 mm^−^^1^
*c* = 15.1892 (11) Å	*T* = 296 K
β = 100.821 (6)°	Prism, colourless
*V* = 1743.4 (2) Å^3^	0.62 × 0.48 × 0.22 mm
*Z* = 4	

### Data collection

**Table:**

Stoe IPDS 2 diffractometer	3958 independent reflections
Radiation source: sealed X-ray tube, 12 x 0.4 mm long-fine focus	3034 reflections with *I* > 2σ(*I*)
Plane graphite monochromator	*R*_int_ = 0.029
Detector resolution: 6.67 pixels mm^-1^	θ_max_ = 27.6°, θ_min_ = 2.3°
ω scans	*h* = −23→23
Absorption correction: integration (*X-RED32*; Stoe & Cie, 2002)	*k* = −8→8
*T*_min_ = 0.881, *T*_max_ = 0.955	*l* = −19→11
10084 measured reflections	

### Refinement

**Table:**

Refinement on *F*^2^	Secondary atom site location: difference Fourier map
Least-squares matrix: full	Hydrogen site location: inferred from neighbouring sites
*R*[*F*^2^ > 2σ(*F*^2^)] = 0.042	H-atom parameters constrained
*wR*(*F*^2^) = 0.110	*w* = 1/[σ^2^(*F*_o_^2^) + (0.0522*P*)^2^ + 0.2998*P*] where *P* = (*F*_o_^2^ + 2*F*_c_^2^)/3
*S* = 1.03	(Δ/σ)_max_ < 0.001
3958 reflections	Δρ_max_ = 0.21 e Å^−^^3^
237 parameters	Δρ_min_ = −0.27 e Å^−^^3^
0 restraints	Extinction correction: *SHELXL97* (Sheldrick, 2008), FC^*^=KFC[1+0.001XFC^2^Λ^3^/SIN(2Θ)]^-1/4^
Primary atom site location: structure-invariant direct methods	Extinction coefficient: 0.0052 (11)

### Special details

**Table:**

Geometry. Bond distances, angles *etc*. have been calculated using the rounded fractional coordinates. All su's are estimated from the variances of the (full) variance-covariance matrix. The cell e.s.d.'s are taken into account in the estimation of distances, angles and torsion angles
Refinement. Refinement on *F*^2^ for ALL reflections except those flagged by the user for potential systematic errors. Weighted *R*-factors *wR* and all goodnesses of fit *S* are based on *F*^2^, conventional *R*-factors *R* are based on *F*, with *F* set to zero for negative *F*^2^. The observed criterion of *F*^2^ > σ(*F*^2^) is used only for calculating -*R*-factor-obs *etc*. and is not relevant to the choice of reflections for refinement. *R*-factors based on *F*^2^ are statistically about twice as large as those based on *F*, and *R*-factors based on ALL data will be even larger.

### Fractional atomic coordinates and isotropic or equivalent isotropic displacement parameters (Å^2^)

**Table:**

	*x*	*y*	*z*	*U*_iso_*/*U*_eq_	
S1	0.82101 (3)	0.21261 (9)	0.49178 (3)	0.0661 (2)	
O1	0.57137 (8)	−0.3003 (2)	0.04586 (9)	0.0678 (5)	
O2	0.69462 (8)	−0.2533 (3)	0.04013 (11)	0.0879 (6)	
N1	0.63001 (7)	−0.0347 (2)	0.11876 (9)	0.0488 (4)	
N2	0.76393 (7)	0.19558 (19)	0.30855 (8)	0.0395 (4)	
N3	0.73011 (8)	−0.0492 (2)	0.38509 (9)	0.0502 (4)	
N4	0.69353 (8)	−0.0842 (2)	0.29879 (9)	0.0506 (4)	
N5	0.78893 (7)	0.3744 (2)	0.27189 (9)	0.0461 (4)	
C1	0.51976 (10)	−0.1911 (3)	0.08396 (12)	0.0528 (5)	
C2	0.44430 (11)	−0.2291 (3)	0.07683 (16)	0.0697 (7)	
C3	0.40518 (11)	−0.0913 (4)	0.11896 (16)	0.0753 (8)	
C4	0.43962 (11)	0.0728 (4)	0.16624 (15)	0.0715 (7)	
C5	0.51655 (10)	0.1116 (3)	0.17325 (13)	0.0599 (6)	
C6	0.55532 (9)	−0.0251 (3)	0.13032 (10)	0.0456 (5)	
C7	0.63901 (11)	−0.1995 (3)	0.06662 (12)	0.0596 (6)	
C8	0.69017 (9)	0.1059 (3)	0.15606 (11)	0.0529 (5)	
C9	0.71518 (8)	0.0672 (2)	0.25402 (10)	0.0420 (4)	
C10	0.77295 (8)	0.1213 (2)	0.39537 (10)	0.0434 (4)	
C11	0.84178 (9)	0.4744 (3)	0.31801 (11)	0.0505 (5)	
C12	0.87066 (8)	0.6636 (2)	0.28379 (11)	0.0449 (5)	
C13	0.85106 (10)	0.7124 (3)	0.19330 (12)	0.0565 (6)	
C14	0.87728 (12)	0.8909 (3)	0.16082 (15)	0.0698 (8)	
C15	0.92305 (12)	1.0222 (3)	0.21815 (18)	0.0753 (9)	
C16	0.94356 (11)	0.9736 (3)	0.30694 (17)	0.0674 (8)	
C17	0.91832 (9)	0.7942 (3)	0.34227 (13)	0.0529 (6)	
C18	0.94257 (14)	0.7470 (4)	0.44018 (16)	0.0802 (8)	
H2	0.42100	−0.34200	0.04520	0.0840*	
H3	0.35370	−0.11010	0.11530	0.0900*	
H3A	0.72590	−0.12990	0.42890	0.0600*	
H4	0.41110	0.16160	0.19460	0.0860*	
H5	0.54000	0.22390	0.20530	0.0720*	
H8A	0.67270	0.24760	0.14660	0.0630*	
H8B	0.73230	0.08710	0.12570	0.0630*	
H11	0.86300	0.42740	0.37500	0.0610*	
H13	0.82000	0.62380	0.15460	0.0680*	
H14	0.86410	0.92290	0.10020	0.0840*	
H15	0.94010	1.14430	0.19640	0.0900*	
H16	0.97520	1.06280	0.34470	0.0810*	
H18A	0.98610	0.82820	0.46430	0.1200*	
H18B	0.95470	0.60270	0.44770	0.1200*	
H18C	0.90240	0.77980	0.47110	0.1200*	

### Atomic displacement parameters (Å^2^)

**Table:**

	*U*^11^	*U*^22^	*U*^33^	*U*^12^	*U*^13^	*U*^23^
S1	0.0839 (4)	0.0758 (3)	0.0346 (2)	−0.0221 (3)	0.0009 (2)	−0.0032 (2)
O1	0.0728 (8)	0.0642 (8)	0.0609 (8)	0.0012 (7)	−0.0013 (6)	−0.0295 (7)
O2	0.0712 (9)	0.1311 (14)	0.0591 (9)	0.0311 (9)	0.0062 (7)	−0.0369 (9)
N1	0.0457 (7)	0.0603 (8)	0.0389 (7)	−0.0010 (6)	0.0039 (5)	−0.0145 (6)
N2	0.0417 (6)	0.0436 (7)	0.0328 (6)	−0.0067 (5)	0.0059 (5)	−0.0014 (5)
N3	0.0569 (8)	0.0546 (8)	0.0386 (7)	−0.0128 (6)	0.0074 (6)	0.0046 (6)
N4	0.0523 (7)	0.0565 (8)	0.0421 (7)	−0.0142 (6)	0.0068 (6)	−0.0025 (6)
N5	0.0530 (7)	0.0443 (7)	0.0399 (7)	−0.0085 (6)	0.0057 (5)	0.0031 (6)
C1	0.0567 (10)	0.0530 (9)	0.0448 (9)	−0.0032 (8)	−0.0005 (7)	−0.0062 (7)
C2	0.0622 (11)	0.0728 (13)	0.0676 (13)	−0.0179 (10)	−0.0044 (9)	0.0032 (10)
C3	0.0509 (10)	0.1009 (17)	0.0711 (14)	−0.0056 (11)	0.0034 (9)	0.0198 (12)
C4	0.0624 (11)	0.0892 (15)	0.0650 (12)	0.0249 (11)	0.0177 (9)	0.0078 (11)
C5	0.0604 (10)	0.0635 (11)	0.0539 (10)	0.0106 (9)	0.0057 (8)	−0.0113 (9)
C6	0.0461 (8)	0.0506 (9)	0.0375 (8)	0.0030 (7)	0.0011 (6)	−0.0051 (6)
C7	0.0599 (10)	0.0753 (12)	0.0400 (9)	0.0120 (9)	−0.0002 (7)	−0.0175 (8)
C8	0.0508 (9)	0.0694 (11)	0.0367 (8)	−0.0118 (8)	0.0036 (6)	−0.0021 (7)
C9	0.0391 (7)	0.0498 (8)	0.0368 (7)	−0.0060 (6)	0.0067 (6)	−0.0036 (6)
C10	0.0455 (8)	0.0486 (8)	0.0363 (7)	−0.0028 (7)	0.0081 (6)	0.0003 (6)
C11	0.0533 (9)	0.0509 (9)	0.0434 (8)	−0.0082 (7)	−0.0010 (7)	0.0060 (7)
C12	0.0433 (8)	0.0419 (8)	0.0495 (9)	−0.0003 (6)	0.0086 (6)	0.0042 (6)
C13	0.0614 (10)	0.0560 (10)	0.0519 (10)	−0.0004 (8)	0.0105 (8)	0.0082 (8)
C14	0.0835 (14)	0.0639 (12)	0.0674 (13)	0.0069 (11)	0.0281 (10)	0.0218 (10)
C15	0.0765 (13)	0.0500 (11)	0.1094 (19)	−0.0025 (10)	0.0431 (13)	0.0200 (12)
C16	0.0590 (11)	0.0476 (10)	0.0968 (17)	−0.0087 (8)	0.0175 (10)	−0.0044 (10)
C17	0.0477 (9)	0.0457 (9)	0.0638 (11)	−0.0027 (7)	0.0066 (7)	−0.0027 (8)
C18	0.0886 (15)	0.0728 (14)	0.0678 (14)	−0.0154 (12)	−0.0146 (11)	−0.0060 (11)

### Geometric parameters (Å, º)

**Table:**

S1—C10	1.6643 (15)	C11—C12	1.463 (2)
O1—C1	1.381 (2)	C12—C17	1.399 (2)
O1—C7	1.369 (2)	C12—C13	1.390 (2)
O2—C7	1.202 (3)	C13—C14	1.374 (3)
N1—C6	1.396 (2)	C14—C15	1.376 (3)
N1—C7	1.355 (2)	C15—C16	1.367 (4)
N1—C8	1.449 (2)	C16—C17	1.390 (3)
N2—N5	1.3944 (18)	C17—C18	1.501 (3)
N2—C9	1.3701 (19)	C2—H2	0.9300
N2—C10	1.3839 (19)	C3—H3	0.9300
N3—N4	1.3716 (19)	C4—H4	0.9300
N3—C10	1.3391 (19)	C5—H5	0.9300
N4—C9	1.2934 (19)	C8—H8A	0.9700
N5—C11	1.253 (2)	C8—H8B	0.9700
N3—H3A	0.8600	C11—H11	0.9300
C1—C6	1.375 (3)	C13—H13	0.9300
C1—C2	1.371 (3)	C14—H14	0.9300
C2—C3	1.369 (3)	C15—H15	0.9300
C3—C4	1.364 (3)	C16—H16	0.9300
C4—C5	1.398 (3)	C18—H18A	0.9600
C5—C6	1.367 (3)	C18—H18B	0.9600
C8—C9	1.493 (2)	C18—H18C	0.9600
			
C1—O1—C7	107.77 (14)	C12—C13—C14	120.31 (17)
C6—N1—C7	109.49 (14)	C13—C14—C15	119.9 (2)
C6—N1—C8	126.61 (14)	C14—C15—C16	120.17 (19)
C7—N1—C8	123.90 (14)	C15—C16—C17	121.7 (2)
N5—N2—C9	118.71 (12)	C16—C17—C18	119.74 (19)
N5—N2—C10	132.76 (12)	C12—C17—C16	117.77 (18)
C9—N2—C10	108.26 (12)	C12—C17—C18	122.50 (18)
N4—N3—C10	114.32 (13)	C1—C2—H2	122.00
N3—N4—C9	103.80 (13)	C3—C2—H2	122.00
N2—N5—C11	118.33 (14)	C2—C3—H3	119.00
N4—N3—H3A	123.00	C4—C3—H3	119.00
C10—N3—H3A	123.00	C3—C4—H4	119.00
C2—C1—C6	122.91 (18)	C5—C4—H4	119.00
O1—C1—C6	109.00 (15)	C4—C5—H5	122.00
O1—C1—C2	128.06 (18)	C6—C5—H5	122.00
C1—C2—C3	116.16 (19)	N1—C8—H8A	110.00
C2—C3—C4	121.81 (19)	N1—C8—H8B	110.00
C3—C4—C5	121.9 (2)	C9—C8—H8A	110.00
C4—C5—C6	116.10 (18)	C9—C8—H8B	110.00
N1—C6—C5	133.14 (17)	H8A—C8—H8B	108.00
N1—C6—C1	105.75 (15)	N5—C11—H11	119.00
C1—C6—C5	121.07 (16)	C12—C11—H11	119.00
O1—C7—N1	107.96 (16)	C12—C13—H13	120.00
O2—C7—N1	128.50 (19)	C14—C13—H13	120.00
O1—C7—O2	123.53 (18)	C13—C14—H14	120.00
N1—C8—C9	110.46 (14)	C15—C14—H14	120.00
N2—C9—C8	122.77 (13)	C14—C15—H15	120.00
N2—C9—N4	111.35 (13)	C16—C15—H15	120.00
N4—C9—C8	125.86 (14)	C15—C16—H16	119.00
N2—C10—N3	102.25 (12)	C17—C16—H16	119.00
S1—C10—N3	126.12 (12)	C17—C18—H18A	109.00
S1—C10—N2	131.61 (11)	C17—C18—H18B	109.00
N5—C11—C12	121.20 (15)	C17—C18—H18C	109.00
C13—C12—C17	120.23 (15)	H18A—C18—H18B	109.00
C11—C12—C13	119.96 (14)	H18A—C18—H18C	110.00
C11—C12—C17	119.81 (15)	H18B—C18—H18C	109.00
			
C7—O1—C1—C2	176.4 (2)	N3—N4—C9—N2	0.12 (17)
C7—O1—C1—C6	−1.5 (2)	N2—N5—C11—C12	−179.86 (14)
C1—O1—C7—O2	−177.25 (19)	C2—C1—C6—C5	0.7 (3)
C1—O1—C7—N1	1.76 (19)	C2—C1—C6—N1	−177.42 (18)
C7—N1—C6—C1	0.53 (19)	O1—C1—C2—C3	−177.6 (2)
C8—N1—C6—C1	−179.11 (15)	C6—C1—C2—C3	−0.1 (3)
C6—N1—C7—O1	−1.43 (19)	O1—C1—C6—N1	0.57 (19)
C8—N1—C7—O2	−2.8 (3)	O1—C1—C6—C5	178.73 (16)
C8—N1—C7—O1	178.22 (14)	C1—C2—C3—C4	−0.8 (3)
C6—N1—C7—O2	177.5 (2)	C2—C3—C4—C5	1.0 (4)
C8—N1—C6—C5	3.1 (3)	C3—C4—C5—C6	−0.3 (3)
C6—N1—C8—C9	74.0 (2)	C4—C5—C6—C1	−0.5 (3)
C7—N1—C8—C9	−105.58 (18)	C4—C5—C6—N1	177.04 (19)
C7—N1—C6—C5	−177.31 (19)	N1—C8—C9—N4	7.5 (2)
C9—N2—N5—C11	−169.40 (15)	N1—C8—C9—N2	−170.82 (13)
C9—N2—C10—S1	−176.79 (12)	N5—C11—C12—C13	−13.5 (2)
N5—N2—C10—S1	−3.0 (3)	N5—C11—C12—C17	166.92 (16)
C10—N2—C9—C8	177.50 (14)	C11—C12—C13—C14	179.29 (17)
N5—N2—C9—N4	−175.91 (13)	C17—C12—C13—C14	−1.1 (3)
C10—N2—N5—C11	17.3 (2)	C11—C12—C17—C16	−179.17 (16)
C9—N2—C10—N3	1.52 (15)	C11—C12—C17—C18	0.9 (3)
C10—N2—C9—N4	−1.08 (17)	C13—C12—C17—C16	1.2 (2)
N5—N2—C10—N3	175.34 (15)	C13—C12—C17—C18	−178.74 (18)
N5—N2—C9—C8	2.7 (2)	C12—C13—C14—C15	−0.1 (3)
C10—N3—N4—C9	0.95 (18)	C13—C14—C15—C16	1.2 (3)
N4—N3—C10—N2	−1.55 (17)	C14—C15—C16—C17	−1.1 (3)
N4—N3—C10—S1	176.89 (11)	C15—C16—C17—C12	−0.2 (3)
N3—N4—C9—C8	−178.40 (15)	C15—C16—C17—C18	179.8 (2)

### Hydrogen-bond geometry (Å, º)

**Table:**

*D*—H···*A*	*D*—H	H···*A*	*D*···*A*	*D*—H···*A*
N3—H3*A*···O2^i^	0.86	2.03	2.856 (2)	162
C4—H4···N4^ii^	0.93	2.52	3.387 (3)	155
C8—H8*B*···O2	0.97	2.58	2.924 (3)	101
C11—H11···S1	0.93	2.48	3.2159 (18)	136
C3—H3···*Cg*2^iii^	0.93	2.94	3.635 (2)	132

Symmetry codes: (i) *x*, −*y*−1/2, *z*+1/2; (ii) −*x*+1, *y*+1/2, −*z*+1/2; (iii) −*x*+1, *y*−1/2, −*z*+1/2.
